# Knowledge, attitudes and practices of contraception by HIV positive women followed in a Cameroon region with high illiteracy rate: a cross sectional study

**DOI:** 10.11604/pamj.2015.20.143.5252

**Published:** 2015-02-17

**Authors:** Elie Nkwabong, Véronique Minda, Joseph Nelson Fomulu

**Affiliations:** 1 Department of Obstetrics & Gynecology, University Teaching Hospital, Yaounde & Faculty of Medicine and Biomedical Sciences, University of Yaounde I, Cameroon; 2 Faculty of Medicine and Biomedical Sciences, University of Yaounde I, Cameroon

**Keywords:** HIV positive women, knowledge, attitude and practices of contraception, Far North Cameroon

## Abstract

**Introduction:**

To evaluate the knowledge, attitude and practices of contraception by HIV positive women.

**Methods:**

This cross-sectional descriptive study was carried out in the Maroua Regional Hospital (Cameroon) from September 1^st^, 2012 to February 28^th^, 2013. All HIV positive women aged between 15 and 49 years who were received in the HIV clinic were recruited. The variables recorded included maternal age, number of living children, marital status, religion, the educational level, and the use of antiretroviral treatment (HAART), the knowledge, attitude and practice of contraception. Analyses were done using SPSS 18.0. Fisher exact test was used for comparison. The level of significance was P < 0.05.

**Results:**

A total of 200 HIV positive women were recruited and 98% knew at least one method of contraception The need of a contraceptive method was present in 84% of HIV positive women, as soon as the women were ≥30 years (OR 2.6, 95%CI 1.3-4.9), on HAART (OR 2.8, 95%CI 0.8-9.2), divorced (OR 1.7, 95%CI 0.8-3.7), had ≥3 living children (OR 1.2, 95%CI 0.6-2.4) and when the women were educated (OR 1.2, 95%CI 0.6-2.4). The rate of condom use was 50.7%.

**Conclusion:**

The knowledge of contraception as well as the contraception need among HIV positive women was high in this region despite high illiteracy rate. Therefore, all contraceptive methods should be made available to these women. Towards these women and their partner(s), more emphasis should be made on the systematic condom use even when using other contraceptive methods (dual protection).

## Introduction

The HIV pandemic rate may continue to increase because its prevalence is increasing due to increased life expectancy, although its incidence is reduced by highly active antiretroviral treatment (HAART) [1]. The appearance of new cases is due mainly to high risk sexual behaviors [2], although counseling about abstinence, faithfulness to one partner or the use of barrier methods (condoms) to prevent new cases is continuously being done. According to UNAIDS, 34 million inhabitants worldwide were HIV positive in 2011 with sub-Saharan African countries harboring 68% of cases [3]. Seventy percent of new cases of HIV infection in 2011 occurred in sub-Saharan African countries [3]. In Cameroon 5.6% of women were HIV positive in 2011 [4]. Many beliefs affect attitudes of HIV positive women towards practices of contraception. Indeed, some women believe that HIV positive women should not procreate so as not to infect their babies [5], while others think that pregnancy status might be associated with poor obstetric outcomes or might alter mother′s health in all HIV positive women [6]. Moreover, even among HIV positive women not desiring to be pregnant, condom use is not systematic in spite of continuous counseling [7, 8]. Henceforth, the knowledge, attitude and practices of contraception by HIV positive women might not be well understood or met. The aim of this study therefore was to evaluate the knowledge, attitude and practices of contraception by HIV positive women in order to revise the counseling that is being given to them.

## Methods

This cross-sectional descriptive study was carried out in the Maroua Regional Hospital (far North Cameroon) from September 1^st^, 2012 to February 28^th^, 2013. All HIV positive women aged between 15 and 49 years who were received in the HIV clinic (clinic for follow-up of HIV positive women) were recruited. In this unit, women receive knowledge on contraception, they are counseled about hygiene and they are advised to use condom during each sexual intercourse. They are asked to report to the HIV clinic in case of any complaint. HAART was prescribed to eligible women: CD4 count less than 350 cells/ml, or WHO stages 3 to 4 diseases when CD4 count was unavailable. After an informed consent form was obtained from each woman, the variables recorded on an anonymous pretested questionnaire form with anonymity and confidentiality by the principal investigator included maternal age, number of living children, marital status, religion, the educational level, the use of HAART, the frequency of sexual intercourse, knowledge, practice and attitudes towards contraception. This study was approved by the national ethics committee. The necessary sample size was calculated as a minimum of 127 women. Analyses were done using SPSS 18.0. Fisher exact test was used for comparison. The level of significance was P < 0.05. Results are presented as mean ± standard deviation (SD) for quantitative data and frequencies for qualitative data. **Footnote:**the formula to calculate sample size is N = P×(1-P)×(Z_α_/d)^2^ where P is the prevalence of HIV infection among women in Cameroon (5.6%), Z_α_=1.96 corresponding to a type I error of 5% and d = 0.04 is the degree of precision.

## Results

A total of 200 HIV positive women were recruited. Maternal ages ranged from 18 to 49 years with a mean of 31.6 ± 6.3 years. The number of living children varied between 0 and 13 with a mean of 3.3 ± 2.2. Regarding educational level, 61 women (30.5%) had never been to school while 95 (47.5%) went to primary school, 36 (18%) to secondary school and eight (4%) had university education. Concerning marital status, the majority was either married or divorced (Table 1). Muslims represented the majority (140 women or 70%) followed by Christians (48 or 24%) and animists (12 or 6%). A total of 188 women (94%) were on HAART, 138 (69%) were sexually active with a frequency of sexual intercourse between one per day to one per month (Table 2). Of the 138 sexually active women, only 70 women (50.7%) were using condoms during each sexual intercourse. A total of 196 women (98%) knew at least one method of contraception and 168 women (84%) had a positive attitude towards the use a contraceptive method now or later in their life. The main reasons for wanting a contraceptive method was that pregnancy could aggravate their infection (37%), the lack of financial means to carry a pregnancy to term (19%) and the unmarried status (19%) (Table 3).

**Table 1:** Marital status of women

Marital status	N (%)
Married	67 (33.5)
Divorced	65 (32.5)
Widow	44 (22)
Single	24 (12)
**Total**	**200 (100)**

**Table 2:** Frequency of sexual intercourse

Average frequency	N (%)
Every day	19 (14)
Thrice weekly	14 (10)
Twice weekly	30 (22)
Once weekly	63 (45)
Once every two weeks	8 (6)
Once per month	4 (3)
**Total**	**138 (100)**

**Table 3:** Reasons for needing a contraceptive method among the 168 women

Reasons	N (%)
Poor health status	62 (37)
Lack of financial means	32 (19.0)
No husband (widow or divorced)	32 (19.0)
Not to infect the future baby	14 (8.4)
No more baby needed	11 (6.5)
Birth spacing	6 (3.6)
Others*	11 (6.5)
**Total**	**168 (100)**

* Not to infect the partner, partner decision, or personal reasons

Prevention of infection of the partner or of other sexually transmissible infections or re-infection as reason for desiring a contraceptive method was rarely mentioned (Table 3). This was confirmed by the fact that only 50.7% of sexually HIV positive women were using condom during sexual intercourse. Among women aged ≥30 years, the need of a contraceptive method was expressed by 87 women as against 64, while for those aged <30 years, 19 women wanted to take a contraceptive method as against 34. Odds ratio (OR) for desiring a contraceptive method for women aged ≥30 years compared to those <30 was 2.6 (95%CI 1.3-4.9, P = 0.0039). Regarding the number of children, among women with ≥3 living children, the need of a contraceptive method was found among 92 women as against 30, while for those with <3 children, 55 women wanted to take a contraceptive method as against 23. OR for desiring a contraceptive method for women with ≥3 children compared to those with <3 children was 1.2 (95%CI 0.6-2.4, P = 0.51). Concerning antiretroviral treatment, among women on HAART, the need of a contraceptive method was revealed in 139 women as against 49 while for those not on HAART, six women wanted to take a contraceptive method as against six who refused. OR for wanting a contraceptive method for women on HAART was 2.8 (95%CI 0.8-9.2, P = 0.09). Regarding the level of education, among women who have never been to school, the need of a contraceptive method was observed among 43 women as against 18, while for those who had at least a primary school education, 104 women wanted a contraceptive method as against 35. OR for wanting to use a contraceptive method for women who went to school was 1.2 (95%CI 0.6-2.4, P = 0.6). Regarding the marital status, among divorced women, the need of a contraceptive method was expressed by 50 women as against 15 while for those who were married, 44 women wanted to use a contraceptive method as against 23. OR for needing a contraceptive method for divorced compared to married women was 1.7 (95%CI 0.8-3.7, P = 0.18). Concerning religion, among Muslims, the need of a contraceptive method was expressed by 109 women as against 30, while for Christians 39 women wanted to take a contraceptive method as against 10 who refused. OR for desiring a contraceptive method for Muslims was 0.9 (95%CI 0.4-2.0, P = 1).

## Discussion

Our study was carried out in an area where Muslims were more represented and where there was a high level of analphabetism. Indeed, 70% of the women were Muslims with 30.5% of women who have never been to school. In this region of Cameroon, little importance is given to women′ education who usually get married earlier, sometimes before 14. The government and the community leaders should fight against precocious marriage and promote women education in this region so that they could actively take part in decisions making. Despite these facts, 98% of the women knew at least one contraceptive method and 84% needed a contraceptive method sooner or later. Since the study was carried out where diagnosed HIV positive women are being followed, the immunity status was deficient in 94% of them. Some were already taking HAART according to the B protocol (zidovudine, lamuvidine and nevirapine or efavirenz). In spite of the depressed immunity status of women, 69% of them were sexually active. Obviously, HAART improved their physical condition to an extent that sexual activity was possible. This improvement has already been shown by others [9]. The main reason why these women on HAART needed a contraceptive method was the fact that pregnancy could alter their immunity. In our series, regular condom use during sexual intercourse was practiced by only 50.7% of sexually active HIV positive women. The low rate of condom use in our series might be due to high rate of analphabetism (30.5%), women not understanding the need of using it even when somebody is already infected. Moreover, some women might not inform their partner of their HIV positive for fear of being abandoned. Finally in this region, only men take (important) decisions in a family. He might refuse to use a condom even if the woman requested it. Low rates of condom use among HIV positive women using another form of contraception was noticed by some [7, 8, 10]. Some authors observed in their series that dual protection was used only by 3.5% of women [11] but others noticed dual protection among 13.5% of women with condom being used by 81.5% of them [12]. This shows that we can improve the rate of condom use with more emphasis especially among men. In this region only community leaders have influence on men. We should, therefore, implicate them in the fight against HIV pandemic. The mean maternal age of our patients was 31.6 years, though the youngest was 18 years. The mean number of living children per woman was 3.3, though the highest number was 13. This is favored by early marriage and the non use of family planning methods. Early marriage exposes girls not only to pregnancy but also to HIV infection. Once more, the government and the community leaders should take concrete actions. Given that the majority of these women (84%) needed a contraceptive method sooner or later, we interested ourselves to the factors that might have influenced them to take this decision. We found that women on HAART had an odds ratio (OR) of 2.8 (95%CI 0.8-9.2) of desiring a contraceptive method perhaps because of the impaired immunity status, since most of these women said that pregnancy could reduce their immunity. Some authors found that women on HAART needed more contraception than women not on HAART [13]. OR for women ≥30 years for wanting a contraceptive method was 2.6 (95%CI 1.3-4.9, P = 0.0039). This might be explained by the fact that at this age, the wanted number of children was achieved. This has also been found by others [11]. Moreover, these women might like to take care of their health rather than continuing to procreate. Divorced women needed a contraceptive method more than married women (OR 1.7, 95%CI 0.8-3.7) even though there was no statistically significance difference. This has already been noticed elsewhere [5]. This might be explained by the fact that they might lack either financial means (unemployment is frequent among women in our series who have to depend on their partner to survive), or moral support from a husband to carry a pregnancy to term and to take care of a baby. Furthermore, they might be afraid that pregnancy could alter their immunity. Contraception is not usually accepted by some men because they are afraid that their partners may become unfaithful given that these women will be virtually protected against pregnancy. OR for women with 3 living children or more wanting to use a contraceptive method was 1.2 (95%CI 0.6-2.4). Some researchers too found that contraception was used mostly by HIV positive women who have great number of children [11]. Women who have been to school had an OR of 1.2 of accepting a contraceptive method (95%CI 0.6-2.4), though not statistically significant. Education might help them understanding better the need of a contraceptive method. We found that religion had almost no influence on the need to use a contraceptive method (OR 0.9, 95%CI 0.4-2.0). We observed in this study that contraception needs was high (84%) among HIV positive women. The majority of these women (66.5%) was widow, single or abandoned (divorced). If they have a new partner, they may need to procreate. Henceforth, short or long lasting contraceptives should be given to these women rather than irreversible contraception since high regret rate with irreversible contraception has been observed by some authors [14].

## Conclusion

This study found that the knowledge of contraception was high in HIV positive women. The need of a contraceptive method was present in 84% of HIV positive women followed in Maroua Regional Hospital as soon as women were ≥30 years, on HAART, divorced, had ≥3 living children especially if these women have been to school. We should, therefore, integrate family planning into HIV clinics. For better implementation of decisions taken by government, the latter and community leaders should fight against precocious marriages and promote women education, given that important decisions in the family are taken by men in this region and only community leaders have an influence on them. The rate of condom use was only 50.7% mostly as a contraceptive method rather than a method for preventing dissemination of HIV infection or re-infection. Henceforth, community leaders should be involved in the sensitization of men too on condom use by HIV positive women or couple. Though all contraceptive methods should always be made available to them, especially short or long lasting contraception, towards these women more emphasis should be made by health care providers on systematic condom use when using other contraceptive methods (dual protection).
